# *M89V Sialic Acid Acetyl Esterase (SIAE)* and All Other Non-Synonymous Common Variants of This Gene Are Catalytically Normal

**DOI:** 10.1371/journal.pone.0053453

**Published:** 2013-01-07

**Authors:** Vasant Chellappa, Kendra N. Taylor, Kathryn Pedrick, Carlos Donado, Ilka Arun Netravali, Khaleda Haider, Annaiah Cariappa, Natasha F. Dalomba, Shiv Pillai

**Affiliations:** Massachusetts General Hospital Cancer Center, Harvard Medical School, Boston, Massachusetts, United States of America; Tulane University, United States of America

## Abstract

Catalytically defective rare variants of *Sialic acid Acetyl Esterase (SIAE)* have previously been linked to autoimmunity. Studies presented here confirm that the M89V SIAE protein and all other products of common variant alleles of *SIAE* are catalytically normal. Although overexpressing transfected non-lymphoid cells secrete small amounts of SIAE that can associate with the cell surface, normal human lymphocytes do not exhibit cell surface SIAE, supporting genetic evidence in mice that indicates that this protein functions in a lymphocyte intrinsic manner. Analyses of the plasma proteome also indicate that SIAE is not secreted in vivo. A re-analysis exclusively of catalytically defective rare variant alleles of *SIAE* in subjects in which this gene was completely sequenced confirmed an association of *SIAE* with autoimmunity. A subset of catalytically defective rare variant *SIAE* alleles has previously been typed in a large genotyping study comparing a diverse group of disease subjects and controls; our re-analysis of this data shows that catalytically defective alleles are enriched in disease subjects. These data suggest that *SIAE* may be associated with autoimmunity and that further study of catalytically defective rare variant *SIAE* alleles in terms of autoimmune disease susceptibility is strongly warranted.

## Introduction

Sialic acid acetyl esterase (SIAE) removes acetyl moieties from the 9-OH position of sialic acid, and thus permits α2,6 linked sialic acid on N-glycans on B cell glycoproteins to interact with CD22/Siglec-2, a sialic acid binding lectin that can inhibit B cell antigen receptor signaling [1]–[3]. Mice with an engineered defect in *Siae* exhibited enhanced B cell receptor (BCR) signaling, B cell differentiation defects that are consistent with enhanced BCR signaling, circulating anti-chromatin antibodies and immune complex deposits in the kidney [1].

We showed in overexpression studies that SIAE can be secreted and decorate the surface of over-expressing transfected non-lymphoid cells presumably by binding to some extracellular component but the physiological relevance of secretion and cell surface expression of this protein was not critically evaluated by in vivo studies [1]. Clearly this protein must function in a post Golgi compartment since α2–6 linked sialic acids moieties are added and acetylated in the 9-OH position in the trans-Golgi. Our previous studies involving the reconstitution of Rag-1 mutant mice with bone marrow from wild type and *siae* mutant mice had suggested that this gene functions in a B lymphocyte intrinsic manner [1]. Although SIAE is expressed in many different tissues, this result indirectly argued against an in vivo role for secreted SIAE.

Catalytically defective heterozygous rare variants of *SIAE* were shown by us to be linked to autoimmune disorders [4], [5]. Although overexpressed wild type SIAE protein was detected in the supernatants of transfected 293T cells, all disease related catalytically defective SIAE proteins expressed in 293T cells exhibited a failure of secretion potentially because these proteins are partly misfolded and are thereby retained in the endoplasmic reticulum (ER).

A catalytically normal common variant of *SIAE*, c.265 A>G (M89V) *SIAE* is seen in heterozygous form in approximately 10% of control subjects (Table 1), and in our original studies was identified in homozygous form in 6/923 subjects with autoimmunity and 0/654 controls [4]. In the course of overexpression studies, a recreated human M89V SIAE protein was shown to be catalytically active, was incapable of inhibiting the activity of the wild type SIAE protein, but was poorly secreted when overexpressed. Although we had assumed that *M89V SIAE* may be defective in spite of being catalytically normal, it has become important to determine whether secretion of wild type SIAE occurs in vivo, and if human lymphocytes express SIAE on the cell surface, in order to determine if secretion alone is a valid assay for SIAE function and to thus assess if the M89V allele is indeed in any way defective.

**Table 1:** Non-synonymous common variants of *SIAE* in subjects of European ancestry.§

Coding change	Sequence Variant	Genotype	Surolia et al (4) Frequency (%)n = 1571	EVS Frequency (%)	dbSNP Frequency (%)n = 1089
G64S	c.190G>A	G/A	0.6365	0.7385*	3.33
K71R	c.212A>G	A/G	1.655	12.0615*	9
M89V	c.265A>G	A/G	8.466	7.9077*	2.7
M89V	c.265A>G	G/G	0.5092	0.3024**	
A467V	c.1400C>T	C/T	0.7002	4.3385*	2.3

§ The subjects from Surolia et al. (4) include controls and autoimmune subjects of European ancestry. EVS (Exome variant server) data comprises unannotated American subjects (disease status is unknown) of European and African ancestry and is current as of June 20th 2012. Variants reported in both African-Americans and European-Americans are marked with a single asterisk (*n = 6500). Variants seen only in European-Americans or only in African-Americans are marked with double (**n = 4299) and triple asterisks (***n = 2201) respectively. The dbSNP data is not ethnically stratified and was derived from the 1000 genomes project.

The most detailed publicly available catalogue of exome sequencing (not annotated to distinguish between controls and disease subjects, but stratified by ancestry), is the NHLBI Exome Variant Server (http://evs.gs.washington.edu/EVS/). The examination of this unannotated data indicates that about 0.3% of Caucasians are homozygous for *M89V SIAE*. Examination of larger sets of subjects by genotyping has revealed that homozygosity of *M89V SIAE* is frequently observed in controls [6], [7]. In a large genotyping study [7], examining a mixture of autoimmune, inflammatory and allergic disorders, it was claimed that *SIAE* was not linked to autoimmunity. Our re-analysis of the data from that study on an allele by allele basis (presented below) indicates that catalytically defective *SIAE* alleles are indeed enriched in subjects with disease. We also examined the catalytic activity of all common variants of *SIAE* to determine if any common variant should be considered in terms of disease susceptibility.

## Materials and Methods

### Ethics Statement

All human studies were approved by the Institutional Review Board at Massachusetts General Hospital.

### Site-directed Mutagenesis of Human and Murine *SIAE* cDNA

Full-length C-terminal Flag-tagged human and murine *SIAE* cDNAs were targeted for PCR based mutagenesis using Pfu Turbo DNA polymerase (Agilent). The *K71R* and *A467V SIAE* variants were re-created on a human *SIAE* cDNA in a pcDNA3.1 based mammalian expression construct. *M89V* and the two rare murine *Siae* variants, *C196F* and *Q335P* (murine equivalent of human *Q309P SIAE*) were re-created in a murine *Siae* cDNA. Primers (5′ to 3′) for site-directed mutagenesis were designed based on ENST00000263593 (human) and ENSMUST00000002007 (mouse). The PCR products were digested with Dpn I restriction enzyme (Agilent) and transformed into TOP10 chemically competent *E. coli* cells (Invitrogen). Clones harboring the desired single nucleotide change were verified by DNA sequencing.

### Assays for *SIAE* Enzyme Activity

Assays for SIAE enzyme activity have been reported by us previously [4]. Briefly, each of the *SIAE* variants (K71R, A467V, M89V, C196F and Q335P (murine equivalent of human Q309P)) were transfected transiently in HEK 293T cells, in parallel to cDNA’s encoding wild type (WT) and S127A (catalytically inert) proteins. Cell lysates were immunoprecipitated with monoclonal anti-Flag antibodies (Sigma) and catalytic activity of immunoprecipitated SIAE was assayed using 4-methylumbelliferyl acetate (USB). Equivalent amounts of cell lysate were immunoprecipitated for the enzyme activity assay, and for quantification of the protein by a quantitative Western blot assay on the LI-COR Odyssey imaging system, using an IR Dye 800 CW labeled Goat anti-mouse IgG as the secondary antibody.

### Metabolic Labeling and Pulse Chase Studies

HEK 293T cells transfected with cDNAs encoding *WT, K71R* and *A467V SIAE* were starved for 1 h in methionine/cysteine-free medium and labeled with 0.5 mCi of ^35^S-methionine for 10 min. Cells were either lysed immediately (10 min) or after 2 and 4 hrs of chase with an excess of complete medium. Lysates were immunoprecipitated with anti-Flag antibodies and protein A sepharose beads (Sigma). Proteins were separated by SDS/PAGE (8%) and revealed by autofluorography.

### Characterization of an Anti-*SIAE* Antibody by Immunoprecipitation and Flow Cytometry

293T human embryonic kidney cells, and the BJAB and Ramos B cells lines (ATCC) were used for characterization of a polyclonal anti-SIAE antibody (Novus Biological, H00054414-B01P). 293T cells were transfected with human Flag-tagged *SIAE*. Cell lysates were immunoprecipitated with anti-SIAE (3 µg), isotype control Mouse IgG (3 µg) (BD Biosciences), or anti-Flag (3 µg) antibodies. Immunoprecipitated samples were analyzed by Western blotting using anti-SIAE (1∶1000) (three hours to overnight) or anti-Flag (1∶1000) antibodies and subsequent incubation with anti-Mouse IgG-HRP (1∶20,000) for 1hour. BJAB and Ramos cells were fixed with 2% paraformaldehyde in PBS for 5 minutes at room temperature. The cells were permeabilized with 0.02% Triton-X-100 in 1xPBS-05%BSA (PBS-BSA) for 7 minutes at room temperature. Cells were then incubated with anti-SIAE (3 µg) or isotype control Mouse IgG (3 µg) for 45 minutes at 4°C. The secondary antibody Goat anti-Mouse IgG-FITC (BD Biosciences) was incubated for 40 minutes at 4°C. The cells were washed twice and resuspended with PBS-BSA for flow cytometric analysis.

### Examination of *SIAE* Intracellularly and on the Cell Surface of Human Lymphocytes by Flow Cytometry

Human white blood cells were isolated and examined separately for the presence of surface or intracellular SIAE. Peripheral blood mononuclear cells (PBMC) were isolated from healthy volunteers using hypotonic water lysis, followed by dilution with 10 X HBSS and two washes with PBS-BSA. For extracellular staining, unfixed or fixed PBMC were incubated with anti-SIAE (3 µg) or Isotype control Mouse IgG (3 µg) followed by a secondary antibody, FITC labeled anti-Mouse IgG. CD8-APC-cy7 (BD Biosciences) was used as a control for both extracellular and intracellular staining. For intracellular staining of PBMC the method was the same as described above for the BJAB and Ramos cell lines.

## Results

### A Large Number of Novel Non-synonymous Rare Variants of SIAE have been Discovered by Exome Sequencing

We originally described 19 rare variants of *SIAE* of which 11 were catalytically defective [4). Rare variants are arbitrarily defined as having a frequency below 1% in the population. In a study on primary biliary cirrhosis, three additional rare variants of *SIAE* were identified of which two were shown to be defective [5]. Unannotated exome sequencing studies that are reported on the Exome Variant Server (controls and disease subjects are not distinguished) have revealed a number of non-synonymous rare variants of *SIAE*. A list of *SIAE* rare variants that have been identified so far in subjects of European and African- American ancestry is included in Table 2. The very large and growing number of rare variants of *SIAE* suggests that genotyping studies would be most useful if all possible defective variants are genotyped in order to obtain detailed information regarding susceptibility. Sequencing of all exons of this gene is required in order to accurately identify rare genetic variants. Functional studies on all identified rare variants will most accurately identify those that should be considered relevant to quantify in disease subjects and controls.

**Table 2:** Rare genetic variants of SIAE.§§

	Coding change	SequenceVariant	Surolia et al. (4) Frequency (%) n = 1571	Hirschfield et al. (5)Frequency (%) n = 381	EVS Frequency (%) n = 4299	
1)	A3G	c.8C>G	0.0636		0.5452	***
2)	R27H	c.80G>A			0.0454	***
3)	Y31C	c.92A>G			0.0909	***
4)	N33S	c.98A>G	0.0636			
5)	A45S	c.133G>T			0.0465	**
6)	W48X	c.143G>A	0.0636			
7)	R62H	c.185G>A			0.0233	**
8)	T81M	c.242C>T			0.0454	***
9)	P91R	c.272C>G			0.0154	**
10)	E96K	c.286G>A			0.0909	***
11)	Q101K	c.301C>A			0.0909	***
12)	N107K	c.321C>G			0.0930	**
13)	A147V	c.440C>T			0.0465	**
14)	S158F	c.286C>T			0.0454	***
15)	Q161K	c.481C>A	0.0636			
16)	A171V	c.512C>T			0.1363	***
17)	C196F	c.587G>T	0.191		0.0462	*
18)	F199C	c.596T>G		0.7874		
19)	R201H^nf^	c.602G>A				
20)	R201C	c.601C>T			0.2726	***
21)	T206I	c.617C>T			0.0465	**
22)	P210L	c.629C>T			0.0154	**
23)	G212R	c.634G>A	0.0636		0.0698	**
24)	S228C	c.683C>G			0.0454	***
25)	R230W	c.688C>T	0.0636			
26)	D246N	c.736G>A			0.1817	***
27)	P251L	c.752C>T			0.0233	**
28)	S255A	c.763T>G			0.0233	**
29)	C266G	c.796T>G	0.0636			
30)	Y276H	c.826T>C			0.0233	**
31)	T286M	c.857C>T			0.0154	*
32)	L296F	c.886C>T			0.0233	**
33)	Q309P	c.926A>C	0.0636			
34)	T312M	c.935C>T	0.1273	0.2625	0.0154	*
35)	R314H	c.941G>A	0.0636		0.0454	***
36)	L323F	c.969A>C			0.1363	***
37)	S325P^nf^	c.973T>C				
38)	D334N	c.1000G>A			0.0454	***
39)	R340H	c.1019G>A			0.0909	***
40)	W341X^nf^	c.1022G>A				
41)	G348S	c.1042G>A			0.0454	***
42)	Y349C	c.1046A>G	0.0636			
43)	P356L	c.1067C>T		0.2625		
44)	N357S	c.1070A>G			0.0233	**
45)	L366V	c.1096C>G			0.0233	**
46)	R369K	c.1106G>A			0.0233	**
47)	D370H	c.1108G>C			0.0233	**
48)	S371L	c.1112C>T			0.0233	**
49)	Q382R	c.1145A>G		0.2625		
50)	A385T	c.1153G>A			0.0909	***
51)	R387W	c.1159C>T			0.0233	**
52)	R393C	c.1177C>T			0.0909	***
53)	R393H	c.1178G>A	0.0636			
54)	A394T	c.1180G>A			0.0233	**
55)	F404S	c.1211T>C	0.2546	0.5249	0.0698	**
56)	G419E^nf^	c.1256G>A				
57)	Q428L^nf^	c.1283A>T				
58)	K434T	c.1301A>C			0.0233	**
59)	C443R^nf^	c.1340A>G				
60)	H447R	c.1340A>G	0.0636		0.0454	***
61)	M456T	c.1367T>C	0.0636		0.0454	***
62)	M456I	c.1368G>A	0.0636			
63)	V459I^nf^	c.1375G>A				
64)	Q462R	c.1385A>G	0.0636		0.0698	**
65)	H472Q	c.1416T>A			1.3176	***
66)	R479C	c.1435C>T	0.0636		0.0233	**
67)	G514A	c.1541G>C			0.0233	**

§§ The subjects from Surolia et al. (4) and Hirschfield et al. (5) include controls and autoimmune subjects of European ancestry. EVS (Exome variant server) data comprises unannotated American subjects (disease status is unknown) of European and African ancestry and is current as of June 20th 2012. Rare variants reported in both African-Americans and European-Americans are marked with a single asterisk (*n = 6500). Rare variants seen only in European-Americans or only in African-Americans are marked with double (**n = 4299) and triple asterisks (***n = 2201) respectively.

nf These variants were found in dbSNP and/or 1000 genomes project but frequency data is not available. The dbSNP data is not ethnically stratified and was derived from the 1000 genomes project.

### Pulse-chase Studies Reveal Minimal Secretion of Overexpressed *SIAE* Proteins

**Figure 1:**
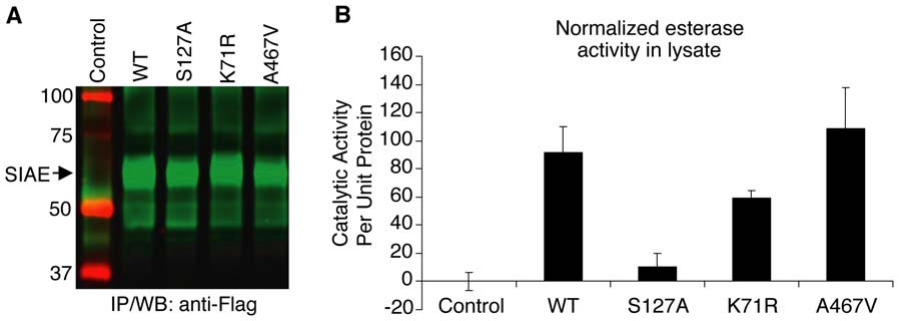
K71R SIAE and A467V SIAE proteins are functionally normal. The *K71R* and *A467V SIAE* variants were re-created by site-directed mutagenesis. Wild type (*WT) SIAE*, a known catalytic site mutant (*S127A SIAE*), and the two *SIAE* common variants were transfected into HEK 293T cells. Half of each cell lysate was immunoprecipitated with anti-Flag antibodies and revealed in a quantitative Western blot assay (A) and the other half was similarly immunoprecipitated and examined for esterase activity, presented following normalization for lysate SIAE protein content (B). Each mutant was separately transfected and analyzed on three occasions. A representative experiment is shown. Error bars reflect esterase assays performed in triplicate in a single experiment.

**Figure 2:**
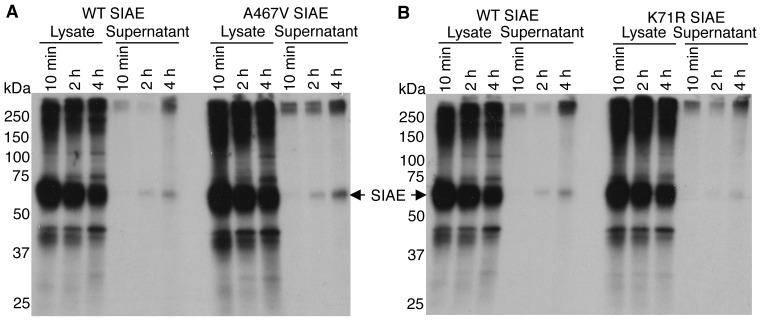
Metabolic labeling and pulse-chase analysis comparing the secretion of wild type SIAE with K71R and A467V SIAE proteins. HEK 293T cells transfected with cDNAs encoding WT, K71R and A467V SIAE were pulse labeled with ^35^S-methionine and lysates and supernatants were immunoprecipitated with anti-Flag antibodies after 10 min, 2 hrs and 4 hrs of chase. Proteins were separated by SDS/PAGE and revealed by autofluorography. The position of molecular weight markers is indicated on the left in kilodaltons (kDa). (A) A comparison of wild type and A467V SIAE proteins. (B) A comparison of wild type and K71R SIAE proteins.

All variants that are at a frequency above the threshold set for rare variants are considered to be common variants. A total of four non-synonymous common variants of *SIAE* were identified in the course of our studies, and all four have also been observed in subsequent exome sequencing approaches (Table 1). We have previously analyzed *G64S SIAE* and *M89V SIAE* [4], both of which encode catalytically normal proteins, though the latter variant was initially assumed to be defective on the basis of defective secretion upon overexpression and this criterion clearly deserves further analysis as discussed in the Introduction. An examination of K71R SIAE and A467V SIAE revealed that they were both catalytically active (Fig. 1), catalytic activity being well above the 50% threshold previously set [4]. We decided to examine the proportion of wild type SIAE, K71R SIAE and A467V SIAE that is secreted in the course of metabolic labeling and pulse-chase studies to indirectly gauge the relevance of protein secretion as an assay for SIAE function. As seen in Fig. 2, over a 4 hour chase period, miniscule amounts of wild type SIAE, K71R SIAE and A467V SIAE were actually secreted suggesting that while the absence of secretion at steady-state may correlate well with a defective ER retained protein in overexpression studies, secretion of SIAE alone may not be a suitable measure of its function.

### *SIAE* is Expressed by Human Lymphocytes but does not Decorate the Cell Surface

**Figure 3:**
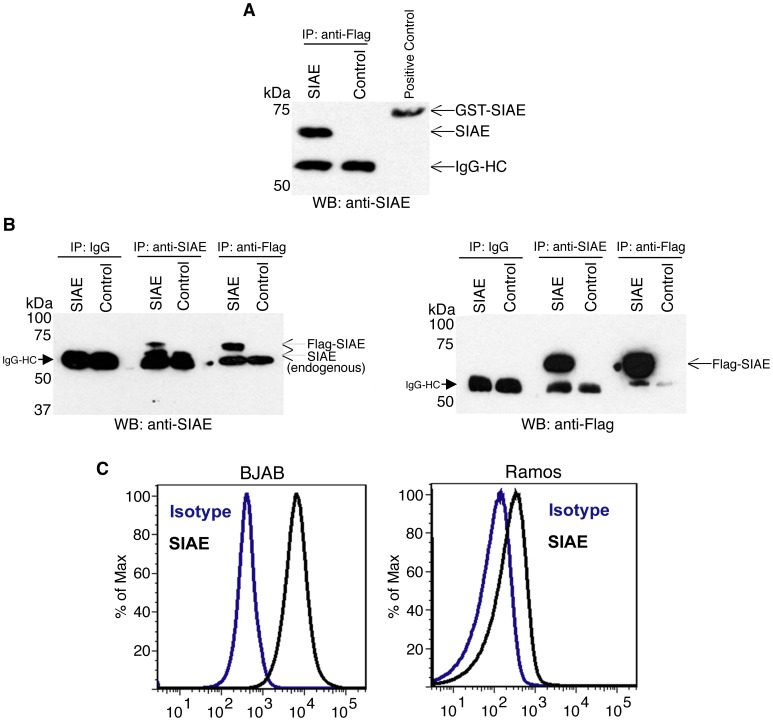
Detection of endogenous and overexpressed SIAE in the native state using a polyclonal anti-SIAE antibody. 293T cells were transfected with Flag-tagged *SIAE* or left untransfected as a negative control. The IgG heavy chain (IgG HC) was seen in all immunoprecipitated samples. (A) Cell lysates were immunoprecipitated with anti-Flag antibodies and analyzed by Western blotting using polyclonal anti-SIAE. An SIAE-GST fusion protein was run in the third lane as a positive control. (B) Cell lysates in both panels were immunoprecipitated with anti-SIAE, IgG and anti-Flag. The left panel displays the use of anti-SIAE for Western blotting. The immunoprecipitated samples in this panel reveal both overexpressed and endogenous SIAE. Immunoprecipitated proteins in the right panel were revealed by an anti-Flag Western blot. (C) Endogenous SIAE levels were further characterized in the B cell lines BJAB (left) and Ramos (right) by flow cytometric analysis. The data are all representative of three independent experiments.

SIAE was originally purified from the liver [8], and it is expressed in cells other than B cells. Detailed proteomic analysis of all plasma proteins as well as of shed human proteins that accumulate at detectable levels in the plasma have been performed. These studies have failed to reveal the presence of even a trace of SIAE in human plasma [9], [10], indicating that SIAE is likely not secreted in vivo. These studies strongly suggest that the secretion of SIAE is likely an in vitro artefact. The cell intrinsic function of this protein in murine B cells is consistent with its expression intracellularly in B lymphocytes. We characterized polyclonal antibodies to SIAE to establish their specificity and to determine whether they were capable of recognizing native protein in immunoprecipitation and flow cytometric studies. As seen in Figures 3A and B, these polyclonal anti-SIAE antibodies can immunoprecipitate overexpressed human SIAE as well as the anti-Flag antibody, and the polyclonal antibodies can also immunoprecipitate endogenous human SIAE. Flow cytometry reveals the presence of endogenous intracellular SIAE in human BJAB and Ramos B cell lines (Fig. 3C).

**Figure 4:**
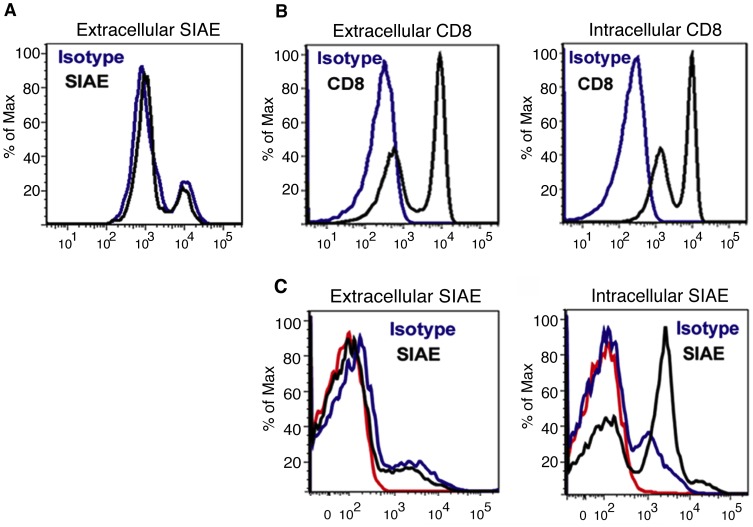
Wild type SIAE is not expressed on the surface of human peripheral blood mononuclear cell (PBMCs). (A) Flow cytometric analysis of SIAE was performed on unfixed non-permeabilized human PBMC demonstrating the absence of SIAE on the surface of any cells in the lymphocyte gate. (B) The analysis of extracellular CD8 on unfixed, non-permeabilized PBMCs (left) and on fixed, permeabilized PBMCs (right) was used as a positive control for extracellular and intracellular staining. (C) SIAE is expressed intracellularly in peripheral blood mononuclear cells. In the left panel, cells were first stained extracellularly with anti-SIAE, washed, and then subjected to fixation and permeabilization prior to analysis. In the right panel, flow cytometry of PBMC for intracellular SIAE was performed on cells that were fixed, permeabilized and then stained with anti-SIAE (black), with an isotype control (blue) or with no antibodies (red).

Flow cytometric studies of human peripheral blood monocnuclear cells (Fig. 4) reveal that SIAE is expressed intracellularly but not on the surface of human white blood cells in the lymphocyte gate suggesting that this enzyme mediates its functions at an intracellular location. This result is consistent with our previous published results indicating that murine Siae functions in a cell-intrinsic manner in B lymphocytes [1].

### Both Murine and Human M89V SIAE Encode Catalytically Normal Enzymes

**Figure 5:**
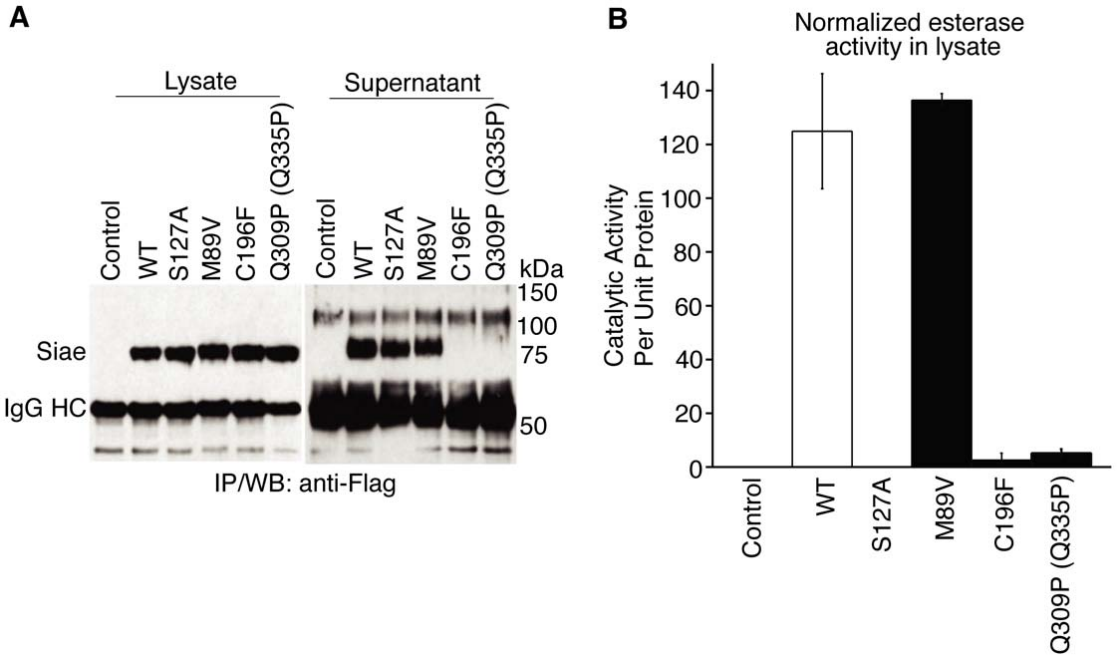
Murine M89V Siae is functionally normal. Murine *Siae* cDNAs encoding WT, S127A, M89V, C196F and Q335P (murine equivalent of human *Q309P SIAE*) were introduced transiently into HEK 293T cells. Half of each lysate was immunoprecipitated with anti-Flag antibodies revealed in a Western blot assay (A, Lysate). The culture supernatants were also subjected to immunoprecipitation with anti-Flag antibodies to reveal the extent of secretion of each Siae variant at steady state (A, Supernatant). The other half of the cell lysate was similarly immunoprecipitated and examined for esterase activity, presented following normalization for lysate SIAE protein content (B). Each mutant was separately transfected and analyzed on three occasions. A representative experiment is shown. Error bars reflect esterase assays performed in triplicate in a single experiment.

To better analyze whether the *M89V SIAE* common variant exhibits an alteration in function, we decided to extend our studies across a species barrier. Human and murine SIAE are not identical (the human protein lacks a 26 amino acid stretch present in rodents) so we determined whether defective human *SIAE* variants are also catalytically defective when re-created in a murine cDNA. All disease related catalytically defective *SIAE* variants tested were defective when re-created in the murine cDNA (see Fig. 5 for *C196F Siae* and the murine equivalent of *Q309P SIAE*). Both catalytically defective disease-related murine proteins were not secreted in these overexpression studies. However overexpressed murine *M89V Siae* (unlike human *M89V SIAE*) was secreted as well as its wild type murine counterpart and like its human counterpart was catalytically normal (Fig. 5). For all these reasons, we believe that only catalytically defective *SIAE* alleles should be compared between disease and controls groups to assess the relevance of this gene is terms of disease susceptibility.

### Catalytically Defective SIAE Alleles may be Linked to Disease

Re-analysis of our original sequencing data examining only the frequency of confirmed catalytically defective *SIAE* alleles in controls and subjects revealed that catalytically defective *SIAE* variants were linked to autoimmunity with an Odds Ratio of 3.51 (95% confidence intervals 1.3684 to 9.0317) and a two tailed p value by Fisher’s exact test of 0.0077 (Table 3). Hunt et al. chose to genotype 8 of the 11 catalytically defective *SIAE* alleles that we had originally described, in a large number of subjects with a variety of diverse autoimmune, allergic, and inflammatory disorders. Interestingly the most frequent but defective rare variant of *SIAE*, *T312M SIAE*, was not genotyped in their studies. This selected subset of catalytically defective rare variants were genotyped in a total of 30, 618 disease subjects from different European cohorts including 22, 718 from the UK. They were compared with a total of 12, 739 control subjects including 8, 274 from the UK. This represents the largest body of genotyping evidence available for catalytically defective *SIAE* alleles – even though a substantially large number of catalytically defective *SIAE* alleles clearly remain to be genotyped. An analysis of this data, allele by allele as well as for all catalytically defective *SIAE* alleles shows a discernible enrichment of catalytically defective *SIAE* alleles with the diverse set of diseases included in the analysis (Table 4), and some alleles such as *SIAE R479C* revealed relatively strong Odds Ratios. Statistical significance could not be revealed in these studies given the low frequencies of rare variants (the two tailed p value even for *R479C SIAE* using Chi square analysis with Yates correction was 0.1995), however these results suggest that it would be premature to assume that catalytically defective *SIAE* alleles are not associated with disease. Keeping in mind that all the catalytically defective rare variants are present at a very low frequency, the inclusion of even a single high frequency catalytically normal common variant can skew the overall analysis and hinder the detection of associations linked to defective rare variants.

**Table 3:** Frequency of catalytically defective rare variants in autoimmune subjects and controls determined by complete sequencing [4].

Autoimmune subjects	Controls	Odds Ratio	P value
16/923	2/648	3.5155 (1.3684 to 9.0317)	0.0077

**Table 4:** Frequency of the subset of catalytically defective *SIAE* variants genotyped by Hunt et al. [7].

Cohort	R479C	F404S	Y349C	R314H	Q309P	G212R	C196F	W48X	All 8
UK Disease	11/22718	0/22718	6/22718	7/22713	4/22716	1/22717	54/22718	2/22717	85/22718
UK Controls	1/8274	1/8274	2/8274	2/8274	4/8274	0/8274	13/8274	0/8274	23/8274
**Odds Ratio**	**4.01**	**–**	**1.09**	**1.28**	**0.36**	**–**	**1.5153**	**–**	**1.3479**
All Disease	11/30618	0/30616	9/30618	9/30618	5/30618	1/30618	61/30618	2/30618	98/30618
All Controls	1/12728	1/12737	2/12727	3/12734	4/12737	0/12738	25/12738	0/12727	36/12739
**Odds Ratio**	**4.57**	**–**	**1.87**	**1.25**	**0.52**	**–**	**1.0145**	**–**	**1.132**

## Discussion

The number of catalytically defective rare variants of *SIAE* originally described by us represents a small subset of the defective rare variants in this gene as revealed by our own subsequent sequence analyses and by exome sequencing studies that have been undertaken subsequent to our original publication. The vast majority of the rare genetic variants of *SIAE* identified by sequencing following our original report represent newly discovered variants, suggesting that the total number of rare variants in this gene will likely be very large. There are currently 67 documented rare variants of *SIAE* and this number is rapidly growing (Table 2).

A very small fraction of overexpressed wild type SIAE is secreted as revealed in pulse-chase studies, and this minimal secretion observed is an in vitro phenomenon of no proven physiological significance. SIAE is not expressed on the surface of human lymphocytes, and has not been detected in human plasma by sensitive means. Secretion of SIAE per se should not be a criterion for the function of this enzyme. Overexpressed murine M89V SIAE is no different from wild type overexpressed protein, and M89V SIAE in both humans and mice is catalytically normal. On these grounds M89V SIAE should be unequivocally excluded from considerations of disease susceptibility. The failure of secretion of overexpressed mutant catalytically defective SIAE proteins in transfected cells is likely a feature of misfolded mutant proteins that fail to egress the ER – and therefore cannot contribute even to the miniscule in vitro escape from the cell. The likelihood that these disease associated mutant proteins are misfolded is currently being explored using circular dichroism spectroscopy.

Re-analysis of our data [4] excluding *M89V SIAE* from analysis, reveals that defective variants of *SIAE* remain associated with autoimmunity, with strong Odds Ratios, but our overall numbers are relatively small compared to those used in the genotyping study of Hunt et al. [7]. Re-analysis of the data of Hunt et al. [7] including only data on the 8 catalytically defective *SIAE* rare variants that they chose to genotype shows that these rare variants are enriched in the diverse disease cohorts they analyzed (Table 4) and these data do not support the notion that there is necessarily a failure of association of catalytically defective *SIAE* alleles with disease.

The analysis of rare genetic variants of any gene will require large scale sequencing analyses followed by functional analyses of novel variants. Large-scale genotyping studies of individual variants may be required in order to attain statistically significant results for any given rare variant allele. Large population studies are currently being carried out with a full sequence analysis of the *SIAE* locus followed by analyses of catalytic function in order to identify a large proportion of defective rare variants of *SIAE* that may be relevant to disease. A wide variety of diseases some allergic, others autoimmune, some neither, were grouped together in the study of Hunt et al. Very large genotyping studies even larger than the one conducted by Hunt et al., but including all known catalytically defective alleles may well provide important information when carried out in well-defined disease populations.

## References

[1] Cariappa A, Takematsu H, Liu H, Diaz S, Haider K, et al (2009) B cell antigen receptor signal strength and peripheral B cell development are regulated by a 9-O-acetyl sialic acid esterase. J Exp Med 206: 125–138. 19103880 10.1084/jem.20081399 PMC2626685

[2] Pillai S, Cariappa A, Pirnie SP (2009) Esterases and autoimmunity: the sialic acid acetylesterase pathway and the regulation of peripheral B cell tolerance. Trends Immunol 30: 488–493. 19766537 10.1016/j.it.2009.07.006 PMC2758290

[3] Pillai S, Netravali IA, Cariappa A, Mattoo H (2012) Siglecs and immune regulation. Annu Rev Immunol 30: 357–392. 22224769 10.1146/annurev-immunol-020711-075018 PMC3781015

[4] Surolia I, Pirnie SP, Chellappa V, Taylor KN, Cariappa A, et al (2010) Functionally defective germline variants of sialic acid acetylesterase in autoimmunity. Nature 466: 243–247. 20555325 10.1038/nature09115 PMC2900412

[5] Hirschfield GM, Xie G, Lu E, Sun Y, Juran BD, et al.. (2012) Association of primary biliary cirrhosis with variants in the CLEC16A, SOCS1, SPIB and SIAE immunomodulatory genes. Genes Immun. 13, 328–335. 10.1038/gene.2011.89 PMC3360983 22257840

[6] Szymanski K, Skorka A, Szypowska A, Bednarczuk T, Ploski R (2011) Functionally defective germline variant of sialic acid acetylesterase (Met89Val) is not associated with type 1 diabetes mellitus and Graves’ disease in a Polish population. Tissue Antigens 78: 214–216. 21615338 10.1111/j.1399-0039.2011.01703.x

[7] Hunt KA, Smyth DJ, Balschun T, Ban M, Mistry V, et al (2012) Rare and functional SIAE variants are not associated with autoimmune disease risk in up to 66,924 individuals of European ancestry. Nat Genet 44: 3–5. 10.1038/ng.1037 PMC3287292 22200769

[8] Higa HH, Manzi A, Varki A (1989) O-acetylation and de-O-acetylation of sialic acids. Purification, characterization, and properties of a glycosylated rat liver esterase specific for 9-O-acetylated sialic acids. J Biol Chem 264: 194365–19442. 2808434

[9] Polanski M, Anderson NL (2007) A list of candidate cancer biomarkers for targeted proteomics. Biomark Insights 7: 1–48. PMC2716785 19690635

[10] Muthusamy B, Hanumanthu G, Reshmi R, Sriranjini S, Suresh S, et al (2005) Plasma proteome database as a resource for proteomics research. Proteomics 5: 3531–3536. 16041672 10.1002/pmic.200401335

